# Age influences the circulating immune profile in pediatric sepsis

**DOI:** 10.3389/fimmu.2025.1527142

**Published:** 2025-01-28

**Authors:** Grace Fisler, Mariana R. Brewer, Omar Yaipen, Clifford S. Deutschman, Matthew D. Taylor

**Affiliations:** ^1^ Cohen Children’s Medical Center, Northwell, New Hyde Park, NY, United States; ^2^ Northwell, Division of Pediatric Critical Care Medicine, New Hyde Park, NY, United States; ^3^ Sepsis Research Laboratory, Feinstein Institutes for Medical Research, Manhasset, NY, United States; ^4^ Northwell, Division of Neonatology, New Hyde Park, NY, United States; ^5^ Department of Surgery, State University of New York (SUNY) Upstate Medical University, Syracuse, NY, United States

**Keywords:** sepsis, infection, immunology, phenotype, pediatrics

## Abstract

**Background:**

The immune response changes as patients age, yet studies on the immune dysregulation of sepsis often do not consider age as a key variable.

**Objective:**

We hypothesized that age would influence the immune response in septic children and that there would be a distinct variation in the immune profile in healthy children and children with either sepsis, uncomplicated infection, or acute organ dysfunction without infection. We characterized the circulating immune profile of children presenting to our tertiary care children’s hospital.

**Methods:**

This investigation was a prospective, observational cohort study that enrolled patients from July 2020 – September 2022. Patients were included if they were < 21 years, admitted to the PICU, and received fluid resuscitation and antibiotics. Peripheral blood mononuclear cells were isolated from samples collected on PICU day 1.

**Results:**

Eighty patients were enrolled. Children with sepsis had more regulatory CD4^+^ T cells and memory CD4^+^ T cells and less CD4^+^IL-10^+^ and CD8^+^T-bet^+^ T cells than healthy children. After *ex vivo* stimulation, sepsis samples had less of a reduction in CD4^+^ T cells producing IL-10 than healthy controls. Memory CD4^+^ T cells and regulatory CD4^+^ T cells were positively associated with age in sepsis alone.

**Conclusion:**

A regulatory T cell failure may contribute to pediatric sepsis pathogenesis. Age is an important variable affecting sepsis-associated immune dysregulation and memory T cells in peripheral circulation correlate with age in sepsis alone.

## Introduction

Sepsis is defined as “life-threatening organ dysfunction caused by a dysregulated host response to infection (1).” Treatment remains limited to antibiotics and supportive care. Targeted therapy of this deadly disorder has focused on modulation of the immune component of the host response. Unfortunately, despite promising data from animal models and results from small human trials, the studied interventions have proven to be ineffective (2, 3). One frequently cited explanation for these confounding and disheartening results is the heterogeneous nature of sepsis (3–7). Recent studies have sought to address this variability by examining immune phenotypes, that is, patterns of expression of a small number of related proteins. The hope is that specific patterns of expression will identify subgroups more likely to respond to a given treatment, as well as cohorts in which a response would not be expected (8–12). Identifying patients for study entry using this approach should enhance the value of data generated in clinical trials. Immune phenotyping of pediatric sepsis is ongoing and ripe for further research (13). Plasma biomarkers can be used to define mortality risk in pediatric sepsis (14–16). Through application of bulk immunology analyses, three immune phenotypes have been described in children with severe sepsis (17, 18). There remains a gap in understanding nuanced immune responses and contributions from small percentages of circulating cells unable to be quantified with bulk techniques that can be assessed by flow cytometry.

The immune response to infection changes from the neonatal period into adulthood. Studies have examined how age affects immune responses in elderly patients; application of these findings has provided insight into the septic response at advanced ages (19, 20). In contrast, while the immune response changes profoundly in children, the effects of the developmental process on sepsis pathobiology has not been examined. We aim to understand how age affects the immunologic changes that occur in pediatric sepsis.

Sepsis is a syndrome defined by organ dysfunction and infection, both of which can occur separately (21–24). Contrasting patients with uncomplicated infection and acute organ dysfunction without infection as patients age may further our understanding of which components of sepsis-associated immune dysregulation are unique to sepsis versus a common process present in severe illness or in uncomplicated infection.

Here, we hypothesized that age would influence the immune response of sepsis and that there would be a distinct variation in the immune profile in healthy children and children with either sepsis, uncomplicated infection, or acute organ dysfunction without infection. To examine our hypothesis, we characterized the peripheral blood mononuclear cell immune profile of children presenting to our tertiary care children’s hospital in the four described cohorts.

## Materials and methods

This investigation was a prospective, observational, non-interventional cohort study that enrolled patients from July 2020 – September 2022. The study was defined by the Northwell Health Institutional Review Board as a minimal risk study (approved 3/27/2020, #20-0191-CCMC). Study protocols were in accordance with the Nuremberg Code and with the Helsinki Declaration of 1975.

Patients met inclusion criteria if they were <21 years old, received fluid resuscitation (≥20cc/kg intravenous crystalloid fluid) and/or vasoactive medications, and received antibiotics, indicating a clinical suspicion for infection. Patients were excluded if admitted after scheduled surgery or if they were COVID PCR+, as institutional restrictions at study onset precluded handling of COVID+ peripheral blood samples. Patients were approached when study personnel were available for informed consent and, when appropriate, assent, within 48 hours of admission. Waste blood was collected and placed immediately into EDTA coated tubes on PICU days 1, 3 (48-72hrs after Day 1) and 7. Clinical variables were collected from the medical record and managed using REDCap electronic data capture tools (25, 26).

The pediatric Sequential Organ Failure Assessment (pSOFA) score was used as a proxy for organ dysfunction (1, 27). Patients were assigned a baseline pSOFA in accordance with their chronic state. A change in pSOFA≥2 was used to identify acute organ dysfunction. This criterion replicates the adult Sepsis-3 criteria with modifications to account for age-specific pediatric and neonatal norms (1, 27). Infection was identified using the following criteria: presence of an organism in blood or urine culture; radiographically confirmed pneumonia; presence of both an organism and neutrophilic infiltration in a specimen obtained from an endotracheal tube or tracheostomy; or viral antigen positivity. In the absence of any of these criteria, a patient prescribed antibiotics for greater than 48 hours had a high clinical suspicion for culture-negative sepsis and was included in the sepsis study cohort. After enrollment was complete, patients were identified as members of one of three cohorts: sepsis (infection + acute organ dysfunction), uncomplicated infection (infection without organ dysfunction), and acute organ dysfunction without infection (AOD). Healthy patients seen in our presurgical testing clinic whose blood was drawn for perioperative testing were recruited to comprise our healthy control cohort.

### Characterization of immune cell subpopulations and plasma cytokine concentrations

Blood was stored at room temperature for up to 12 hours prior to processing. Peripheral blood mononuclear cells (PBMCs) were isolated, counted using a hemocytometer, and frozen in freezing media at -80°C. When sufficient samples were collected, PBMCs were thawed, washed, and stained with fluorochrome-labelled antibodies. An aliquot of PBMCs from each patient was stimulated *ex vivo* as described below.

Cells were stained with LIVE/DEAD fixable viability dye (Life Technologies, Carlsbad CA); samples were plated in duplicate and stained with either a 20-antibody panel for T cell markers or for innate and B cell markers. Intracellular markers (CD68, FoxP3, Granzyme B, IL-10, IL-17A, IFNγ, T-bet, TNFα) were applied after samples were fixed and permeabilized. Stained cells were stored at 4°C for up to 7 days. Flow cytometry was performed on a BD LSR Symphony 23-color cell analyzer (BD Biosciences, San Jose CA). The gating strategy can be referenced in the supplement. Results were analyzed using FlowJo software v10.1 (BD Bioscience, San Jose, CA). Cell counts were determined using the white blood cell count when available from the electronic medical record on the day of sample collection.

Levels of plasma cytokines from Day 1 plasma samples, collected and stored at -80°C, were determined using multiplex ELISA (Eve Technologies, Calgary, Alberta, Canada).

### Response to stimulation

The reserved aliquot of PBMCs noted above was stimulated with phorbol myristate acetate (50ng/ml, PMA) and ionomycin (1.5uM, Io) for three hours at 37°C in 5% CO_2_. The reaction mixture included Brefeldin A (2ug/ml, BD Biosciences, San Jose, CA) to inhibit cytokine secretion to increase flow cytometry sensitivity for intracellular cytokine production (28, 29). After incubation, these cells underwent staining and analysis as described above. All stimulation assays were performed alongside a control without stimulation, as previously described (30).

### Statistical analysis

Categorical data were expressed as frequency (percent) and continuous data as mean (standard deviation) or median (interquartile range). Analyses were performed using GraphPad PRISM 9. We used Kruskal-Wallis test with Dunn’s correction for multiple comparisons to compare subpopulations in different cohorts with healthy controls. To examine relationships between stimulation (yes/no) and sepsis vs healthy cohort, the stimulated samples were subjected to a mixed effect analysis. For analysis of the relationship between subpopulations within a given cohort with subject age, simple linear regression was used. For plasma cytokine analysis, values out of range were assigned the upper or lower limit of the assay. Cytokine values that failed normality based on D’Agostino and Pearson Test underwent natural logarithmic transformation prior to analysis. Cytokine values were analyzed between the four cohorts via one-way ANOVA with Dunnet correction for multiple comparisons of each cohort to the healthy cohort. The sepsis and healthy cohorts were categorized by age (>5 years or ≦5 years) and two-way ANOVA was used to compare plasma cytokine levels. Age of 5 years was used to divide patients by prior to and following adrenarche (31). Plasma cytokine levels that were significantly different on initial one-way ANOVA were included in this analysis.

## Results

A total of 80 patients were enrolled in the study. Characteristics of the four cohorts are detailed in **Table 1**. There were more males enrolled across all cohorts, most notably in the uncomplicated infection group.

**Table 1:** demographics of study patients.

	Healthy (n=33)	Uncomplicated Infection (n=8)	Acute Organ Dysfunction (n=13)	Sepsis (n=27)
Age yrs (median, IQR)	10.5 (6.4-15.0)	10.1 (7.1-18.1)	12.3 (7.5-15.7)	13.1 (2.4-16.2)
Female Sex, n (%)	13 (39)	1 (13)	4 (31)	12 (44)
PICU LOS days (median, IQR)		3.5 (2.6-8.0)	4.5 (2.1-10.3)	7.8 (2.9-15.7)
Hospital LOS (median, IQR)		5.7 (2.8-10.9)	7.7 (3.1-14.4)	14.6 (7.8-25.5)
pSOFA (median, IQR) (1)		0.5 (0-1)	3 (2.5-4)	3 (2-5)
Source of Infection, n (%)				
Bacteremia		3 (38)		8 (30)
Respiratory(2)		1 (13)		5 (19)
Urine		0		2 (7)
Peritonitis		1 (13)		2 (7)
Viral		0		3 (11)
Culture-Negative		3 (38)		7 (26)
Pre-existing conditions, n (%)				
Malignancy		0	2 (15)	6 (22)
Immunodeficiency		0	0	0
Autoimmune Disease		1 (13)	0	3 (11)
Transplant Recipient		1 (13)	0	3 (11)

(1) pSOFA on enrollment less their baseline pSOFA.

Defined as positive endotracheal/tracheostomy culture with evidence of inflammation or pneumonia.

### Plasma levels of cytokines that activate the adaptive immune system were higher in sepsis patients

**Figure 1:**
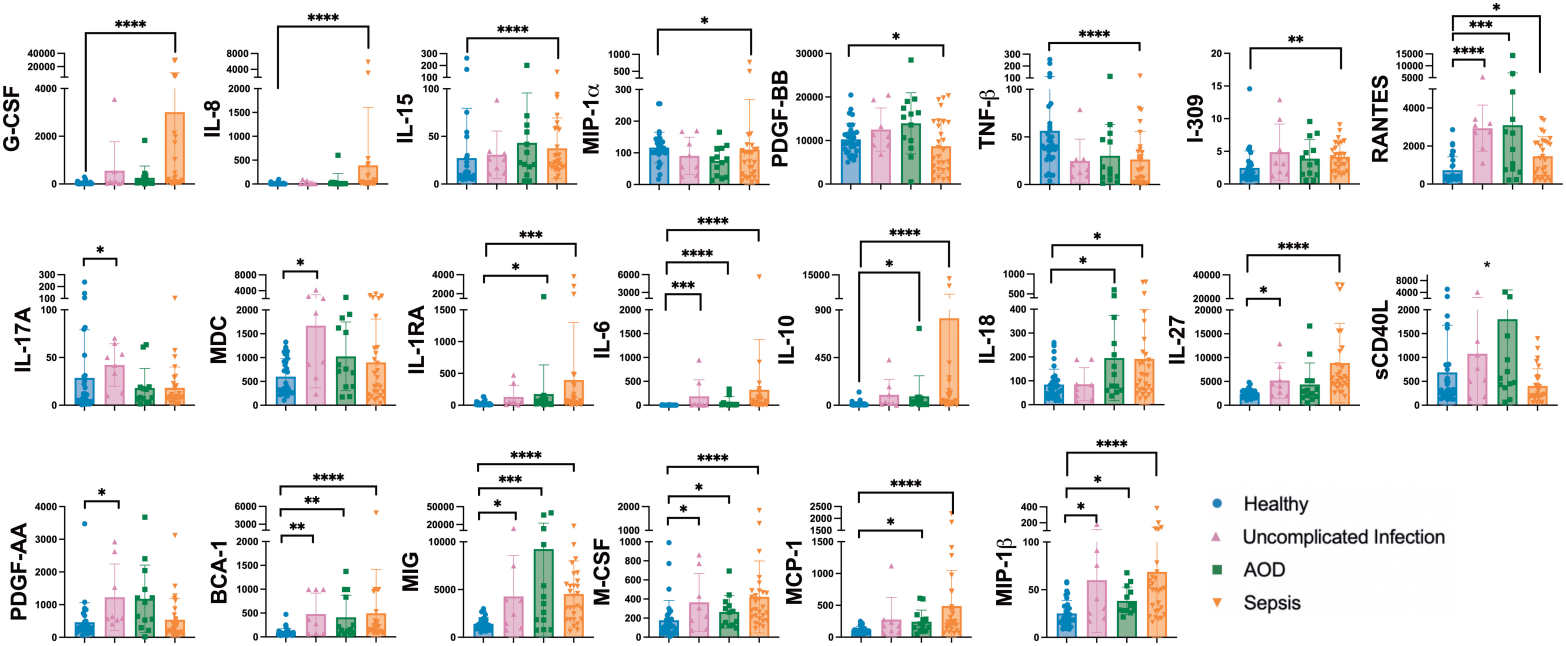
Differences between Plasma Cytokines on Day 1 Between Cohorts Differences in plasma cytokines by Kruskal Wallis with multiple comparisons to healthy cohort. Concentrations are in pg/mL unless otherwise noted. Each dot represents one patient, bars are mean, lines are 95% confidence interval. *p<0.05, **p<0.01, ***p<0.001, ****p<0.0001.

**Figure 1** represents cytokines that differed among cohorts. Several cytokines were uniquely different between healthy and sepsis cohorts. Cytokines known to activate the adaptive immune system were generally higher in sepsis compared to the healthy cohort, including RANTES (p=0.01), G-CSF (p<0.0001), M-CSF (p<0.0001), IL-6 (p<0.0001), IL-8 (p<0.0001), IL-27 (p<0.0001), and MIP-1β (p<0.0001) (32–36). Markers of an IFNγ response were higher in sepsis than in healthy patients, including IL-18 (p=0.02) which increases IFNγ production and MIG (p<0.0001), an IFNγ inducible chemockine (37–40).

IL-10 level was higher in sepsis (p<0.0001) and AOD (p=0.02) compared to the healthy control cohort. IL-10 promotes regulatory T cells, playing a counter-regulatory role to host inflammatory response (41).

Plasma concentrations of IL-17A (p=0.03) were higher in patients with uncomplicated infection than in healthy controls. IL-17A is produced by T cells and induces macrophage and neutrophil production of cytokines which in turn further activates the innate immune response. Levels of this cytokine in the sepsis and healthy cohorts were not statistically distinguishable. Compared to healthy patients, those with uncomplicated infection had higher levels of PDGF-AA (p=0.02), a cytokine known to dampen the immune response, and MDC (p=0.04), a chemokine known to promote neutrophil recruitment (42, 43).

### Cohorts with organ dysfunction had lower proportion of pro-inflammatory monocytes

**Figure 2:**
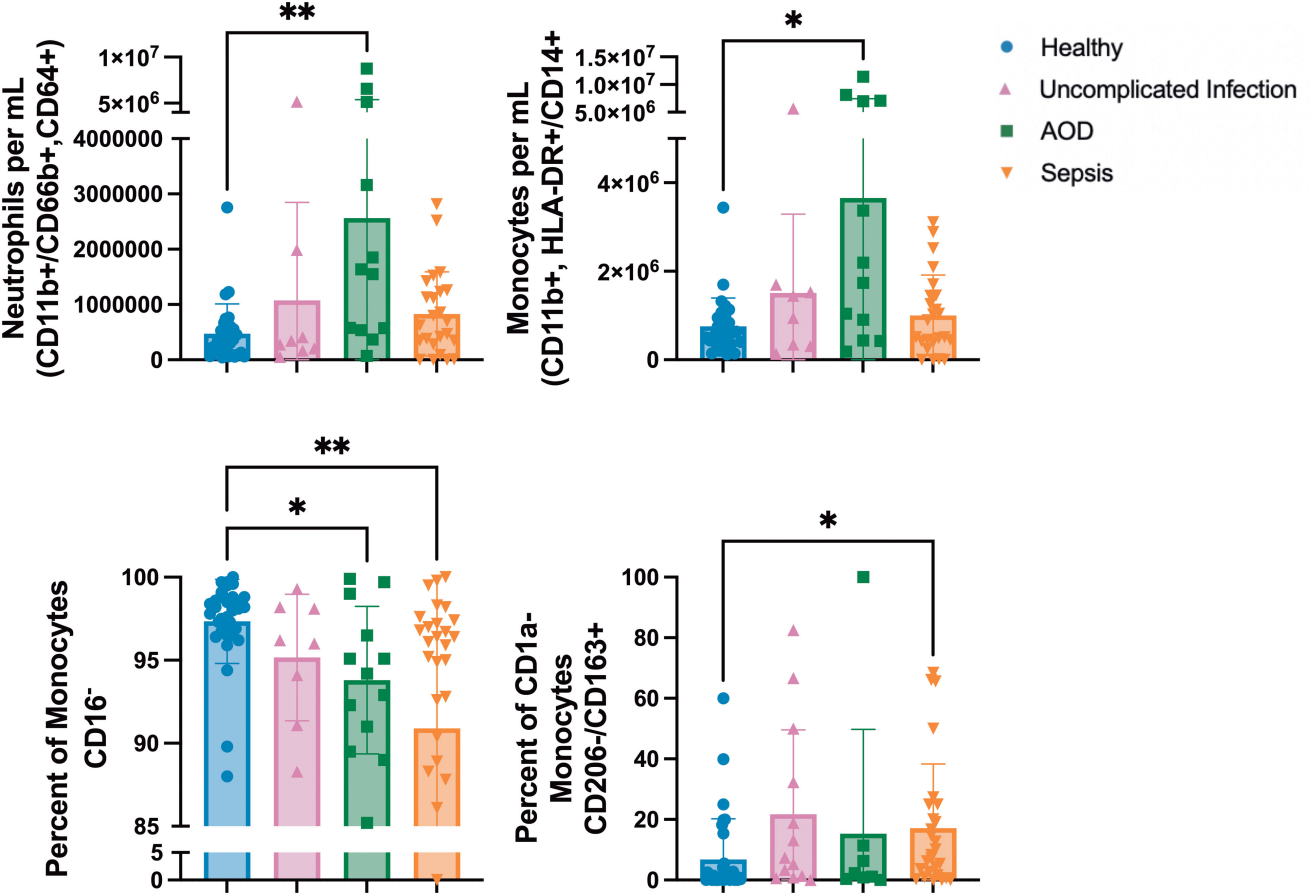
Differences between Innate Subpopulations in those with Sepsis, Acute Organ Dysfunction without Infection, Uncomplicated Infection, and Healthy Children Differences in innate subpopulations by Kruskal Wallis with Dunn correction for multiple comparisons to healthy cohort. Each dot represents one patient, bars are mean, lines are 95% confidence interval. *p<0.05, **p<0.01.

Cytokines may drive immune cellular response or be reflective of those responses. To investigate the cellular immune response to sepsis, PBMCs were examined by flow cytometry. On day 1, total numbers of neutrophils (CD11b^+^, HLA-DR^+^/CD66b^+^, CD64^+^) and monocytes (CD11b^+^, HLA-DR^+^/CD14^+^) were higher in patients with AOD than the numbers in healthy patients (p=0.004 and p=0.02, respectively). Monocytes can be characterized by CD16 expression for inflammatory responses. Healthy patients had higher percentages of pro-inflammatory monocytes (CD16^-^) as a percentage of the peripheral blood mononuclear cell population than did those patients with AOD (p=0.02) or sepsis (p=0.007). Monocytes that were CD206^-^ and CD163^+^ were present in higher proportion in patients with sepsis (p=0.01) (**Figure 2**).

### Sepsis was characterized by less central memory CD4 and CD8 T cells, higher TEMRA CD8 T cells, and lower central memory CD8 T cells

**Figure 3:**
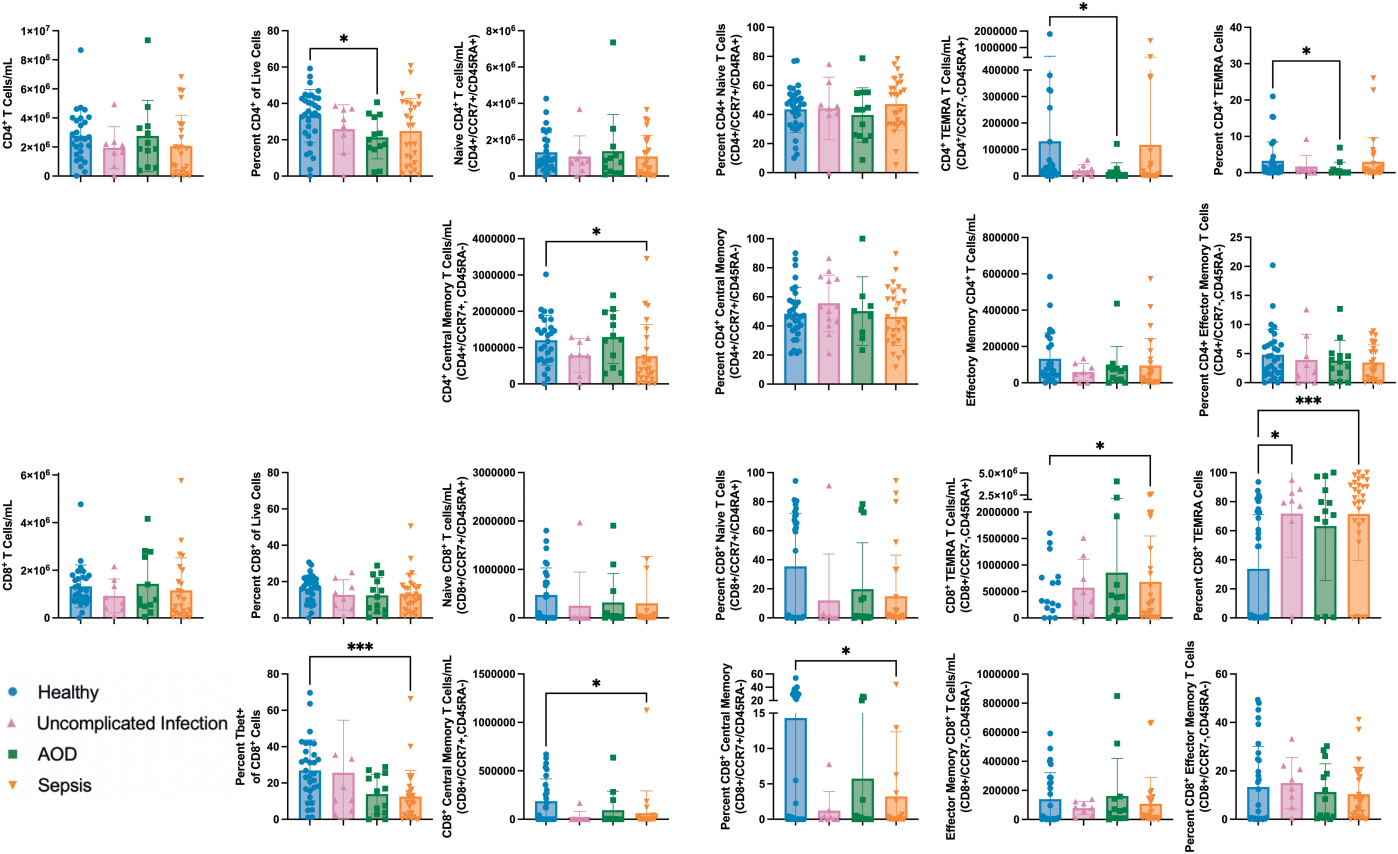
Differences between Naïve and Memory T Cell Subpopulations in those with Sepsis, Acute Organ Dysfunction without Infection, Uncomplicated Infection, and Healthy Children Differences in naïve and memory T cell subpopulations by Kruskal Wallis with Dunn correction for multiple comparisons to healthy cohort. Each dot represents one patient, bars are mean, lines are 95% confidence interval. *p<0.05, ***p<0.001.

There were no differences in the total circulating CD4^+^ or CD8^+^ T cell numbers between cohorts. AOD patients had a lower percentage of CD4^+^ T cells as a percentage of circulating live cells compared to healthy patients (p=0.04). There were no differences in naïve CD4^+^ or CD8^+^ T cells between cohorts. Circulating memory T cells allow for a prompt response to an antigen that the host has previously encountered (44). On Day 1 of admission, there were differences in total circulating numbers of central, effector, and terminal effector memory (TEMRA) CD4^+^ and CD8^+^ T cells (45). Specifically, patients with AOD had less TEMRA (CCR7^-^/CD45RA^+^) CD4^+^ T cells than healthy (p=0.02). The healthy and sepsis cohorts had several differences. There were less central memory (CCR7^+^/CD45RA^-^) CD4^+^ and CD8^+^ T cells in sepsis compared to healthy (p=0.02 and 0.04, respectively). Sepsis patients had a higher number of circulating TEMRA CD8^+^ T cells compared to healthy (p=0.02). The percentage of TEMRA CD8^+^ T cells as a percentage of CD8^+^ T cells was lower in healthy patients than in sepsis (p=0.009) or in uncomplicated infection (p=0.04). The percentage of central memory CD8^+^ T cells was lower in sepsis compared to the healthy cohort (p=0.02). **Figure 3** summarizes these results.

T-bet promotes the differentiation of effector CD8^+^ T cells from central memory T cells (46). In the sepsis cohort, a lower percentage of CD8^+^ T cells expressed T-bet than in healthy controls (12% vs 27%, p<0.001).

### Sepsis patients had a higher proportion of regulatory CD4^+^ T cells, a lower proportion of CD4^+^ T cells producing IL-10, and were less able to alter IL-10 production after ex vivo stimulation

**Figure 4:**
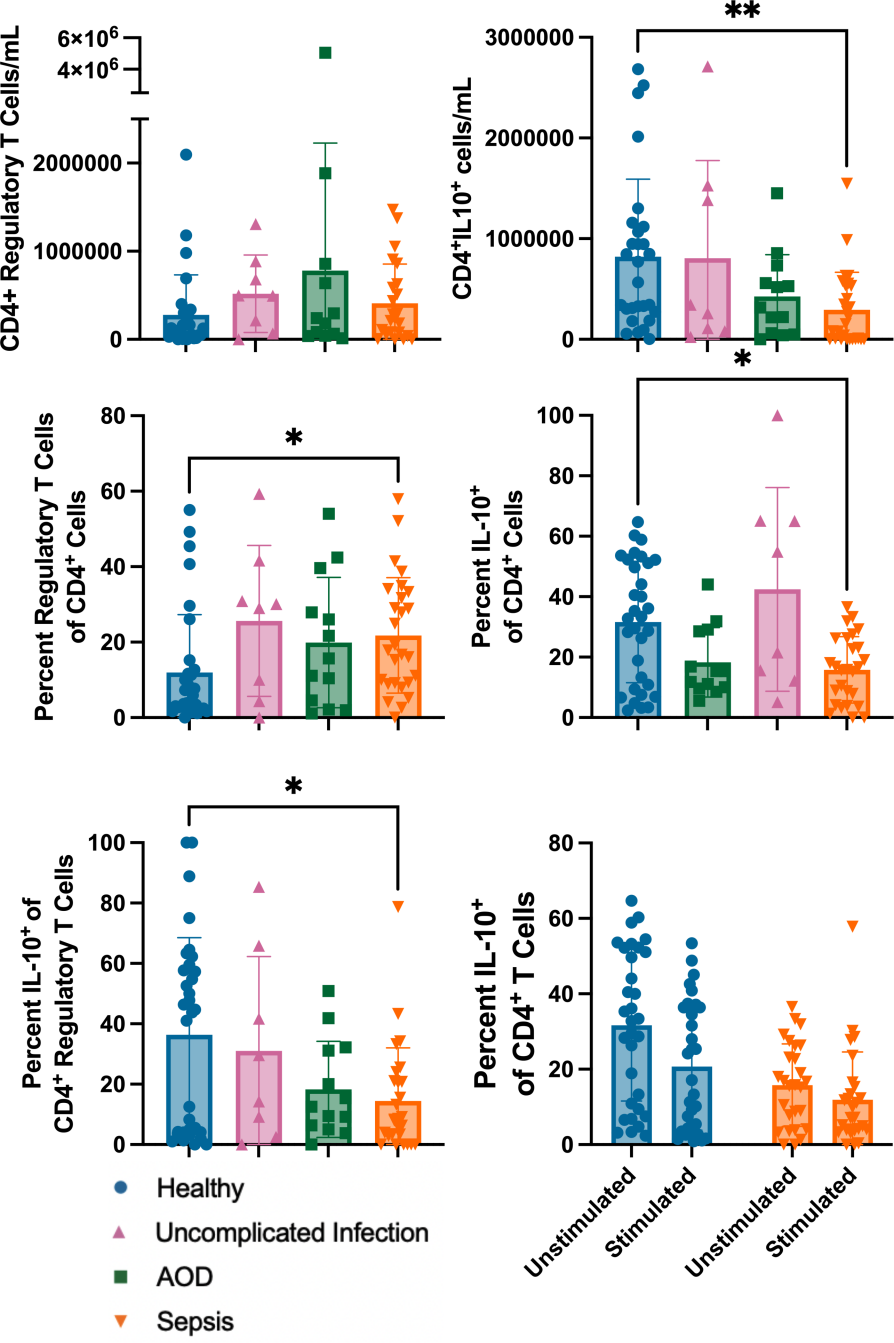
Differences in Regulatory and IL-10 producing CD4+ T Cells at Rest and After *ex vivo* Stimulation Between Cohorts Differences in circulating Regulatory CD4^+^ T Cells and IL-10 producing CD4^+^ T cells between cohorts, compared via Kruskal Wallis with Dunn correction for multiple comparisons to healthy cohort. Also shown is the response to stimulation between the healthy and sepsis cohorts: two-way ANOVA with variables of stimulation (yes/no) and cohort (healthy/sepsis) shows that stimulation and cohort effect percentage of CD4^+^IL10+ cells present. Each dot represents one patient, bars are mean, lines are 95% confidence interval. *p<0.05, **p<0.01.

The sepsis cohort, compared to healthy, had a higher proportion on Day 1 of CD4^+^ T cells expressing regulatory markers (CD45RA^+^/FoxP3^+^) (22% vs 12% of CD4^+^, p=0.01). The sepsis cohort, compared to healthy, had a lower proportion of CD4^+^ T cells producing IL-10 without *ex vivo* stimulation (p=0.01). IL-10 limits the host response and is often produced by regulatory T cells. IL-10^+^ regulatory T cells were present in lower proportions in sepsis patients compared to the proportion present in the healthy cohort (**Figure 4**, p=0.04).

*Ex vivo* cytokine production in response to stimulation represents the cellular capacity to respond to a new challenge. Without stimulation, patients in the sepsis cohort had a lower proportion of CD4^+^ T cells producing IL-10 than did healthy controls. Both healthy and sepsis cohorts had a reduction in CD4^+^IL10^+^ T cells after stimulation; this reduction was more pronounced in healthy patients compared to sepsis patients (stimulation effect 7% of variation p<0.001, cohort effect 18% of variation p=0.001, stimulation by cohort effect 3% of variation p=0.029).

### Proportions of memory and regulatory T cells correlate with patient age in sepsis

**Figure 5:**
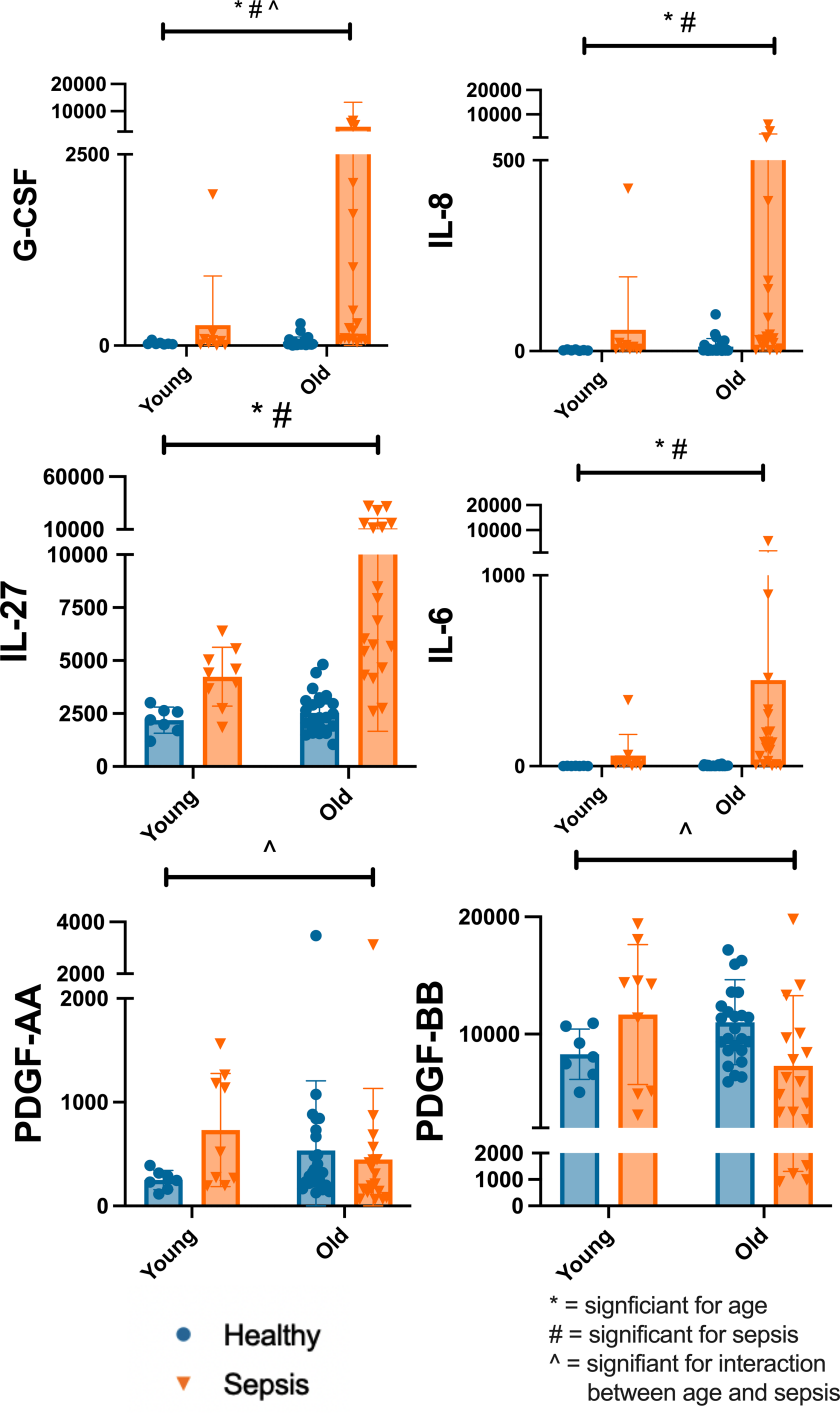
Differences in Select Plasma Cytokine Concentrations Based on Age and Disease State Differences in select plasma cytokine concentrations by Two-Way ANOVA with variables of age (<5yrs, >=5yrs) and cohort (healthy, sepsis). Each dot represents one patient, bars are mean, lines are 95% confidence interval. *significant for age; #significant for sepsis; ^significant for interaction between age and sepsis Significance defined as p<0.05. Young=<5yrs; Old>=5yrs.

In comparing healthy and sepsis cohorts, older age and sepsis were both associated with higher Granulocyte Colony Stimulating Factor (G-CSF) levels. G-CSF level was influenced by both sepsis and age (<5 years vs >=5years) (interaction p=0.007; age p=0.009; sepsis presence p>0.001). Older age and sepsis presence were both associated with higher IL-6 levels. Sepsis presence and older age influenced the level of IL-6 (age p=0.035; sepsis presence p>0.0001). Older age and sepsis presence were both associated with higher IL-8 levels. Presence of sepsis and older age influenced the level of IL-8 (age p=0.032; sepsis presence p>0.0001). Presence of sepsis and older age influenced the level of IL-27 (interaction 3% p=0.056; age p=0.009; sepsis presence p>0.0001). Platelet derived growth factor (PDGF)-AA (p=0.018) and PDGF-BB (p=0.015) were both affected by the interaction between age and sepsis (**Figure 5**). PDGF is produced by several cells including regulatory T cells and serves to modulate T cell behavior, enhancing IL-2 production and decreasing the production of several pro-inflammatory cytokines. This effect on cytokines suggests PDGF serves to dampen the immune response (43, 47, 48).

**Figure 6:**
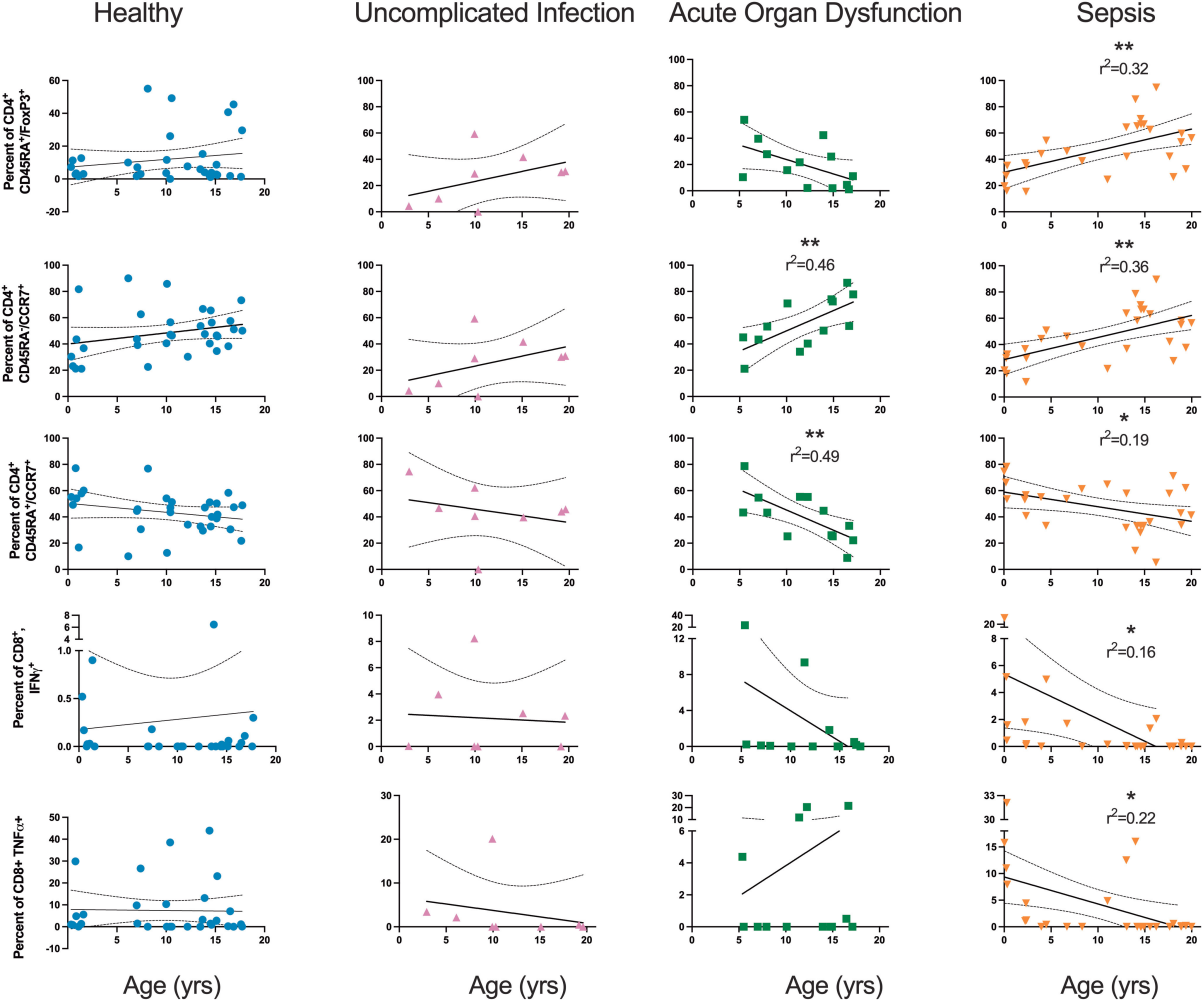
Relationship between Age and Select Subpopulations of the Adaptive Immune System Relationship between age and select subpopulations by simple linear regression. Each dot is one patient. Solid lines are linear regression. Dotted lines are 95% confidence interval. *p<0.05; **p<0.01.

The magnitude of several of the adaptive immune subpopulations on Day 1 samples in the sepsis cohort correlated with patient age (**Figure 6**). In sepsis, age positively correlated with the percent of CD4^+^ cells that were regulatory (CD45RA^+^FoxP3^+^), r^2^ = 0.32, p=0.002. The regulatory CD4^+^ population in the other cohorts had no correlation with age. Age positively correlated with the percent of central memory CD4^+^ (CCR7^+^CD45RA^-^) T cells in sepsis (r^2^ = 0.36, p=0.001) and AOD (r^2^ = 0.46, p=0.01). Healthy patients did not have a correlation with the percent of central memory CD4^+^ T cells and age. Age was inversely correlated with the percent of naïve (CCR7^+^/CD45RA^+^) CD4^+^ T cells in AOD (r^2^ = 0.49, p=0.008) and sepsis (r^2^ = 0.19, p=0.024). Age inversely correlated with the percentage of CD8^+^ T cells positive for IFNγ in sepsis alone (r^2^ = 0.16, p=0.04) and the percentage of CD8^+^ T cells positive for TNFα in sepsis alone (r^2^ = 0.22, p=0.013).

## Discussion

### Main findings

In our study of pediatric sepsis, we found that the magnitudes of the regulatory and central memory T cell responses increase as children age. Age was associated with several differences in peripheral blood cytokine levels, indicating that patients following adrenarche presented with higher levels of pro-inflammatory cytokines. PDGF, a cytokine produced by regulatory T cells to dampen the immune response, was higher in younger sepsis patients (47, 48). Younger patients had a higher proportion of circulating naïve T cells, but also had lower numbers of regulatory and memory T cells. These observations suggest that older children may mount a more pro-inflammatory response to sepsis that may be shaped by T cell immunity and that age is an important variable influencing immune dysregulation in sepsis.

T-bet promotes the differentiation of effector CD8^+^ T cells from central memory cells (46); both CD8^+^ central memory and CD8^+^Tbet^+^ T cells were lower in sepsis patients compared to healthy. Sepsis patients had higher regulatory CD4^+^ T cells but an overall more pro-inflammatory plasma cytokine milieu and higher levels of TEMRA CD8^+^ T cells. Further, CD4^+^IL10^+^ T cells, including IL10^+^ regulatory CD4 T cells, were lower in sepsis compared to healthy patients. These cells were also less functional in sepsis: after stimulating PBMC’s *ex vivo with* PMA/Io, only healthy samples had a statistically significant reduction in CD4^+^IL10^+^ T cells. This differential response suggests the sepsis patients had already mounted a full response and had reduced their IL-10 production, so therefore were unable mount a full response on subsequent *ex vivo* stimulation. These observations suggest either a failure of the regulatory T cell compartment in sepsis or a bias toward a more pro-inflammatory response. This may contribute to overall immune dysregulation.

### Relationship to prior literature

A recent pediatric study found more TEMRA CD4^+^ T cells in sepsis patients, which is comparable to our finding of more TEMRA CD8^+^ T cells in sepsis (49). This study was smaller, limiting comparisons over age, and lacked a healthy cohort. A post-mortem study of sepsis patients demonstrated that spleen and lung tissue showed a decrease in both pro-inflammatory and anti-inflammatory cytokine production and upregulation of inhibitory ligands, though this associated with high levels of several markers of T cell activation. These results suggest multiple mechanisms of immune dysregulation in sepsis characterized by both altered cytokine production and decreased host ability to respond to circulating cytokines (50). Our study on PBMCs and plasma from living sepsis patients showed higher levels of many circulating cytokines, predominantly those that are pro-inflammatory (32–36, 51).

There were less IL-10 producing CD4^+^ T cells in sepsis patients and the ones that were present were less able to respond to *ex vivo* stimulation than their healthy counterparts, which is somewhat congruent with other sepsis observations of a regulatory T cell compartment failure (52). There are also reports of an augmented regulatory T cell compartment in adults with sepsis and septic shock, so perhaps the regulatory T cell compartment failure we observed is unique to children, or perhaps, our early timepoint captured a more acute phase of the sepsis response (52, 53). These studies failed to examine direct T cell intracellular IL-10 production which may further contribute to variation in our observations. This is an important area of future study as a failure of T cell IL-10 production may contribute to the immune dysregulation observed in sepsis.

Understanding immunologic changes over age may provide insight into the differences in immunity that may shape the immune response to neonatal and pediatric sepsis. Following birth, neonates are exposed to a natural environment allowing T cell memory to develop in response to multiple childhood viral and bacterial exposures. Our observation that memory CD4 T cells increase in sepsis as patients age is somewhat congruent with murine studies that show changes in the T cell memory compartment after exposure to a non-sterile environment (54–58).

### Implication of findings

Based on our observations, we propose that a possible mechanism of immune dysregulation in early pediatric sepsis involves a primary failure of the CD4 T cell regulatory response leading to an increased CD8 effector T cell response, altering the monocyte response. Through cell to cell and cytokine interactions, this increased CD8 effector T cell response contributes to systemic immune activation and organ dysfunction. This proposed mechanism requires further testing with mechanistic studies but could support clinical changes in the sepsis response that have been noted as neonates age through childhood to adulthood. Augmentation of the regulatory T cell response in older children represents a potential therapeutic target if our results can be replicated.

We performed several analyses with age as a variable to test our hypothesis that age impacts the immune response to sepsis. Interestingly, we found that age positively correlated with the percent of CD4^+^ central memory T cells in both sepsis and acute organ dysfunction. Age was also inversely correlated with the proportion of naïve CD4^+^ T cells in both sepsis and acute organ dysfunction. These findings suggest that a change in T cell phenotypes contributes to organ dysfunction regardless of the presence of infection. A relationship between age and memory and naïve T cell populations and how these variables influence organ dysfunction requires further investigation.

Our observations suggest that older children mount a more pro-inflammatory response to sepsis and that age is an important variable in the immune dysregulation of sepsis. We have also identified several aspects of the immune response which may drive these differences and warrant future study. Sepsis has a varied clinical presentation and disease course based on patient age, with neonates, younger children, older children, and adults all behaving differently. Further study into the differences in immune dysregulation over age may assist with therapeutic investigations targeting specific immune components based on patient age. Further, given the differing clinical variation between neonates, children and adults, specific immunologic manifestations that are detrimental to one group may be beneficial to another. Our work is the first step in understanding these concepts.

### Limitations and strengths

Sepsis is a heterogenous disease, and in this limited cohort we were unable to sub-analyze our data based on inciting organism, which is likely an important variable (49). Other variables we intend to explore in future studies but were unable to include in this multivariable model include comorbidities and steroid use. Larger patient pools are needed to compare the correlation between age and immune subpopulations between the four study cohorts. We could not control when in the disease course a child presented to the hospital, so we may be including samples of both early and mid-sepsis time points. Only one time point, Day 1, was analyzed because sufficient Day 3 and Day 7 samples were not available due to either quick improvement or early mortality of pediatric sepsis patients.

A notable strength of this study compared to other pediatric observational studies is our unique use of three comparison groups. We observed that those with sepsis, uncomplicated infection, and acute organ dysfunction without infection share several characteristics. We also observed several important differences that may provide mechanistic insights into distinctions between these processes. A further strength of our study was that our healthy cohort was not hospitalized and unexposed to anesthesia, so we believe is representative of the unstimulated immune state (59). Finally, our use of *ex vivo* stimulation allowed us to gain functional information concerning the T cell immune response to pair with our *in vivo* data, extending our ability to comment on potential mechanisms of sepsis biology.

## Conclusions

Our data suggests that age is an important variable affecting sepsis-associated immune dysregulation. Further investigation of changes over age may provide insight into both the adult immune response to sepsis and into differences in immunity that may impact neonatal and pediatric sepsis presentation and responses to therapy. The immune dysregulation in children may be characterized by a failure of the regulatory T cell compartment, and more research is warranted as we move to an era of individualized sepsis therapy. Finally, comparison of the sepsis immune response to uncomplicated infection and acute organ dysfunction may provide further mechanistic insights into these common conditions which impact our patients.

## Data Availability

The raw data supporting the conclusions of this article will be made available by the authors, without undue reservation.
