# Supplementary material for: Age influences the circulating immune profile in pediatric sepsis
Source: Front Immunol. 2025 Jan 28;16:1527142. doi: 10.3389/fimmu.2025.1527142 (PMC11810941; doi:10.3389/fimmu.2025.1527142)
Supplement: Supplementary file 1 [file DataSheet1.pdf]

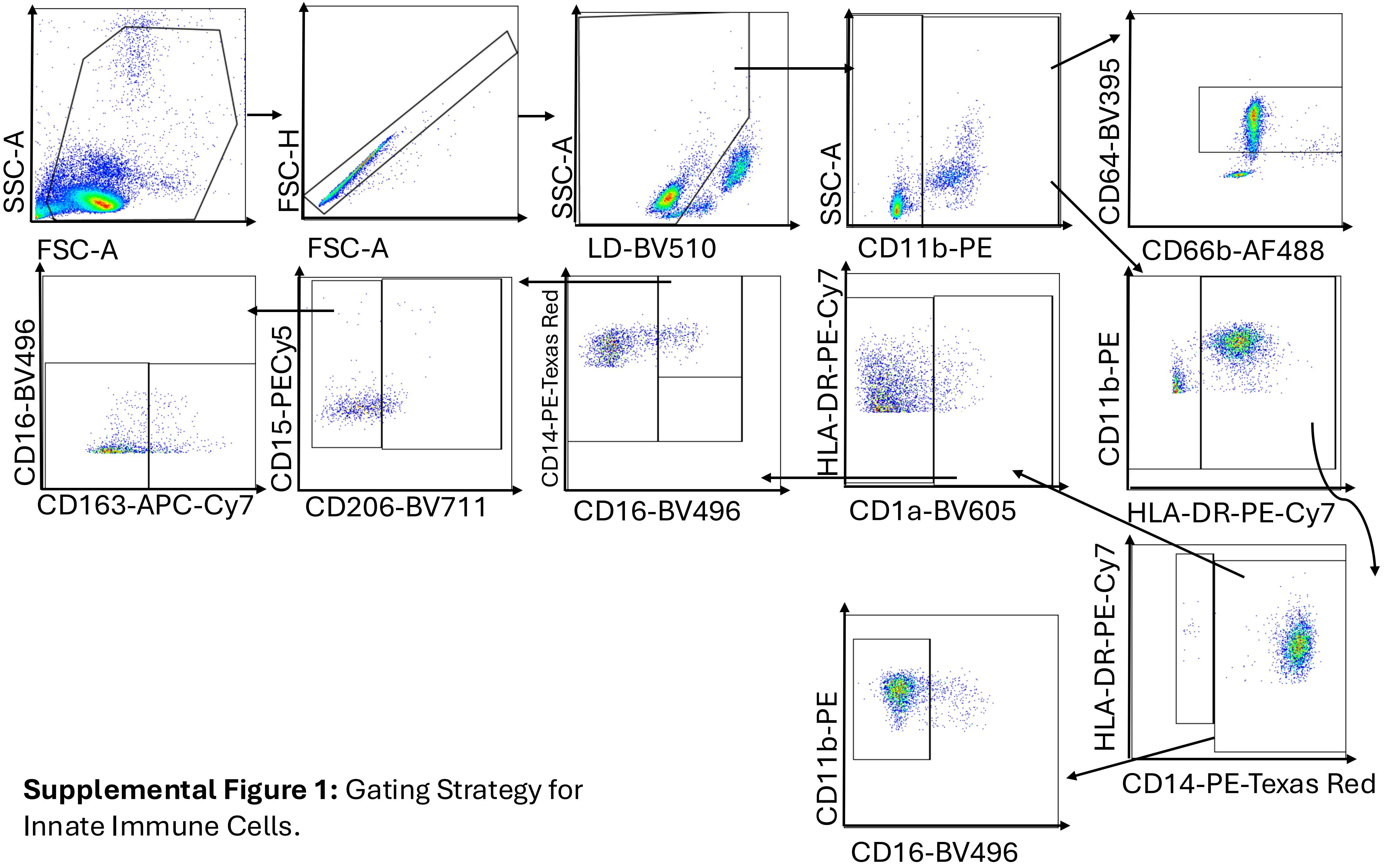

Supplemental Figure 1: Gating Strategy for Innate Immune Cells.

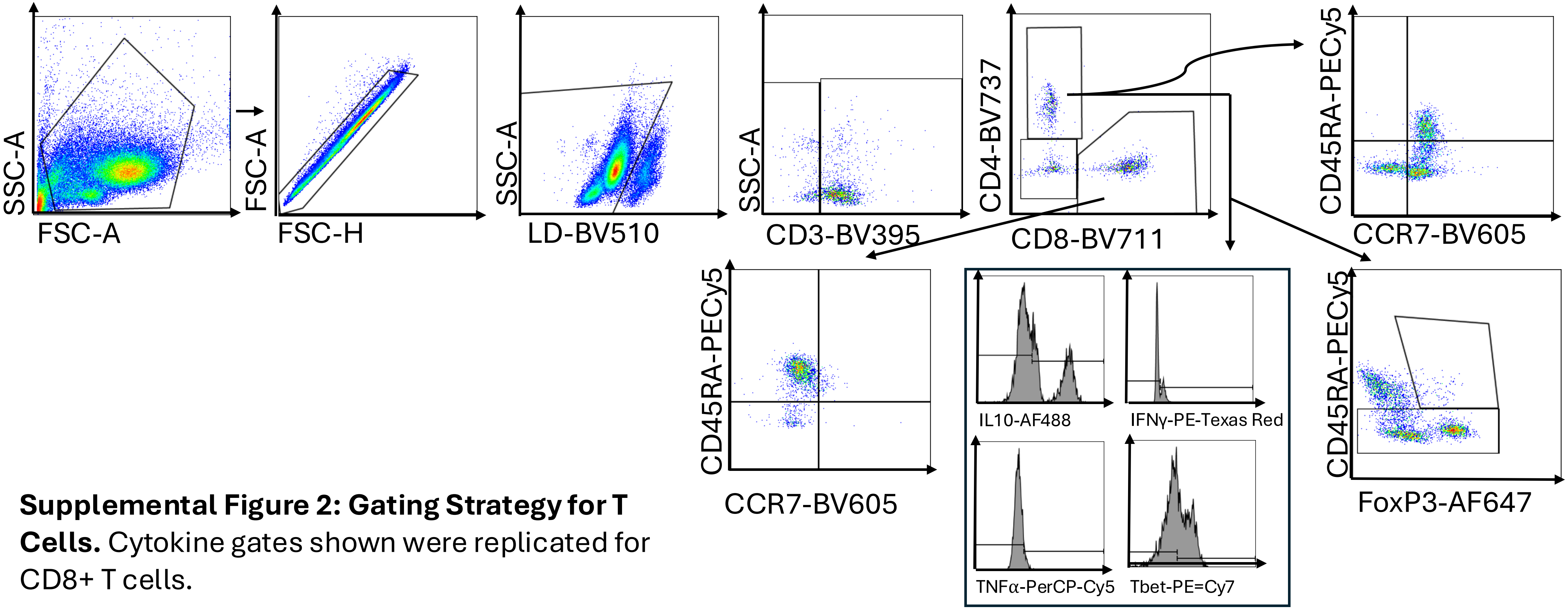

Supplemental Figure 2: Gating Strategy for T Cells. Cytokine gates shown were replicated for CD8+ T cells.

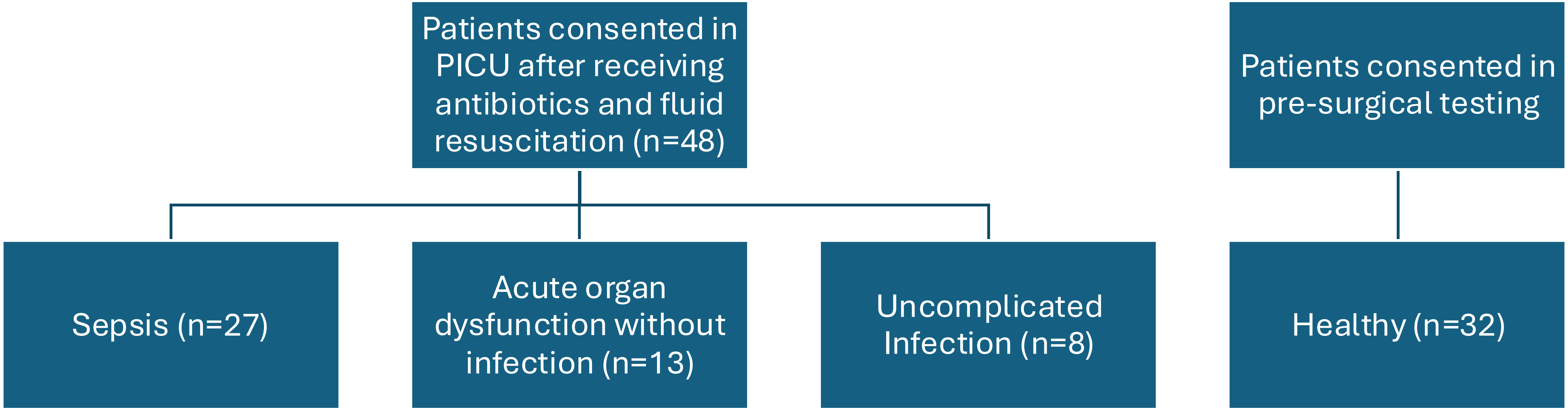

Supplemental Figure 3: Diagram illustrating patient enrollment and cohort composition. *PICU=pediatric intensive care unit*
